# Maxillomandibular Transverse Osteodistraction: A Multidisciplinary Case Report with 30-Month Follow-Up

**DOI:** 10.1155/2020/3856412

**Published:** 2020-01-31

**Authors:** G. Turatti, A. Bruni, M. Savoini, M. Giordano, G. Gerbino

**Affiliations:** ^1^Odontostomatology OU, Martini Hospital, Via Tofane 71, Turin 10141, Italy; ^2^CIR Dental School, Department of Surgical Sciences, Università degli Studi di Torino, Via Nizza 230, Turin 10126, Italy; ^3^Department of Mechanical and Aerospatial Engineering (DIMEAS), Politecnico di Torino, C.so Duca degli Abruzzi 24, Turin 10129, Italy; ^4^Division of Maxillofacial Surgery, Department of Surgical Sciences, Università degli studi di Torino, Città della Salute e delle Scienza Hospital, Corso A.M. Dogliotti 14, Turin 10126, Italy

## Abstract

**Aim:**

To describe a multidisciplinary treatment to correct a severe II class malocclusion with reduced both maxillary and mandibular transverse dimensions and dental crowding. *Case Report*. A 17-year-old young woman presented with an increased overjet complaining chiefly of forwardly placed upper front teeth and unpleasant smile aesthetics. The patient facially exhibited a gently convex profile, severe mentalis strain on lip closure, and dark buccal corridors. The intraoral assessment indicates Class II molar relationship bilaterally, mandibular and maxillary anterior crowding, and narrow shape of upper and lower arches. The cephalometric evaluation of the lateral radiograph of the skull evidences a skeletal Class II with a reduction of lower face height. Based upon the diagnostic records and consultation with the patient, surgically assisted expansion of both arches using bone-borne distractors, comprehensive orthodontic treatment, and combined jaw surgery was planned.

**Results:**

This approach permitted achieving most of the desired objectives in approximately 30 months. The follow-up records 30 months after treatment conclusion showed a stable occlusion. No complications were clinically and radiographically noticeable during the follow-up.

## 1. Introduction

*Transverse deficiencies* are a common finding among populations [1]: in selected patients, concurrent osteodistraction of both arches represents a viable option [2–4] to treat severe maxillomandibular transverse deficiency and dental crowding.

Historically, the mandibular arch dimension has always been considered immutable.

The synostosis of the mandibular symphysis occurs during the first year after delivery, and the use of orthodontic devices (e.g., lingual arches [5], functional appliances [6], Schwarz plates, and archwires [7]) has proved inadequate to gain more than 6-8 mm in the lower arch [8, 9]. Several methods exist to overcome this limit (e.g., through interproximal enamel reduction, extracting teeth, or even using bone anchorages to lower the lateral sectors [8]). However, the stability of these treatments is often unpredictable and can result in adverse side effects [10].

Distraction osteogenesis is the biological process of new bone formation between bone segments that are gradually separated by incremental traction after an intentional surgical bone fracture [10].

The first *Mandibular Symphyseal Distraction Osteogenesis* (MSDO) was performed by Guerrero in 1997 [11] and subsequently described under various nomenclature, e.g., Mandibular Midline (Osteo-) Distraction (MMD) and Transmandibular Symphyseal (Osteo-) Distraction (TMSD).

This treatment modality, compared to the abovementioned ones, improves aesthetics and function, shortens treatment time, and appears stable over time [11].

The fusion of midpalatal suture has a variable timing: it occurs generally after the age of 15 [12].

*Surgically Assisted Rapid Palatal Expansion* (SARPE), also mentioned as *Surgically Assisted Rapid Maxillary Expansion* (SARME), is a widely accepted procedure for skeletally mature patients with maxillary transverse deficiency.

The first *SARPE* was performed by Brown in 1938 [13, 14] to prevent unwanted effects of orthopedic or orthodontic expansion in skeletally mature patients (e.g., buccal tipping of posterior teeth, extrusion, periodontal membrane compression, buccal root resorption, alveolar bone bending, fenestration of the buccal cortex, palatal tissue necrosis, pain, and instability of the expansion [15]) by surgically releasing the closed sutures resisting the expansion forces.

The most frequent indications for a simultaneous bimaxillary osteodistraction, mentioned as *Maxillomandibular Transverse Osteodistraction* (MMTOD), are severe (maxillary-) mandibular transverse deficiency, severe (maxillary-) mandibular anterior crowding [11], uni- [16] and bilateral buccal crossbite, impacted anterior teeth with insufficient space and hugely tipped teeth [17], and obstructive sleep apnea syndrome [18].

In this case report, the focus is on the orthodontic and surgical procedure, the distractor devices, the distraction sequence, the skeletal and aesthetic outcome, the stability after a 30-month follow-up, treatment-related difficulties, and complications.

## 2. Case Report

In September 2013, a 17-year-old female came to the Department of Orthodontics at the Martini Hospital in Torino with the chief complaint of prominent upper teeth and crooked mandibular and maxillary incisors with difficulty in maintenance of oral hygiene.

### 2.1. History

She reported CCE (common childhood exanthemas) when she was a child. She was in good general health, without any allergies. The patient's oral health was good. Before treatment, complete orthodontic and radiographic documentation was requested. No previous orthodontic treatment was performed; no TMJ problems were reported. The first step was to conduct thorough extraoral and intraoral examinations.

### 2.2. Assessment

#### 2.2.1. Extraoral Assessment

**Figure 1:**
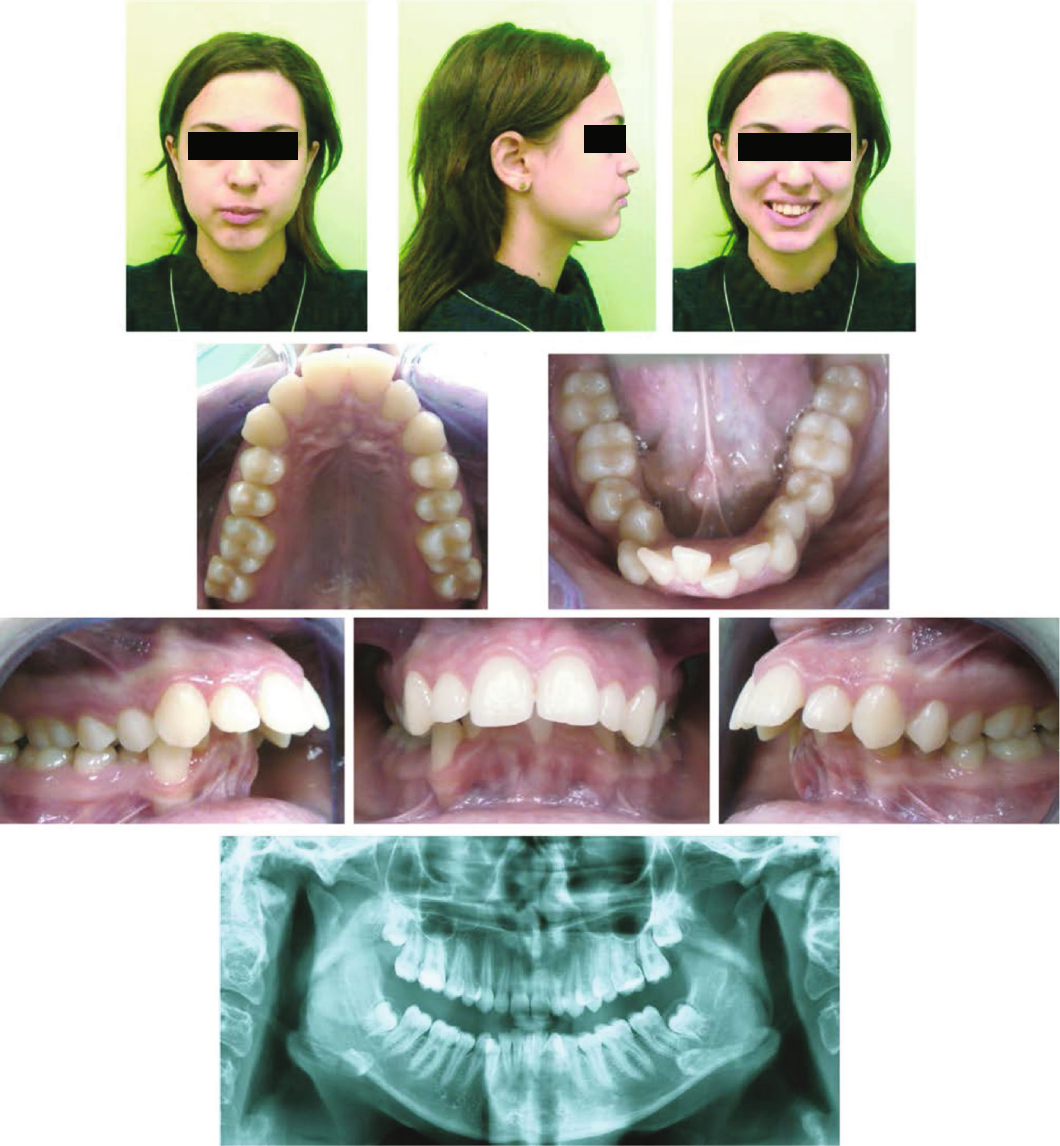
Extraoral photographs (T0), intraoral photographs (T0), and orthopantomogram (T0); T0=pretreatment.

During the extraoral clinical examination, a mandibular retrusion and severe mentalis strain on lip closure were verified; the profile was reasonably convex; and the nasolabial angle was moderately closed. The face shape was oval with moderate mandibular asymmetry to the right side; smile arch appeared to be flattened with an excessive amount of gingival display during forced smiling; and dark buccal corridors were found (Figure 1).

#### 2.2.2. Intraoral Assessment

Intraorally, a narrow shape of the upper arch and the lower arch, a bilateral Class II molar and canine relationship, increased overjet (OVJ: 12.7 mm) and overbite (OVB: 7.2 mm), a dental crowding in both dental arches (slight in the upper, severe in the lower), and deviation of the superior midline to the right of 4 mm and inferior midline to the right of 6 mm were detected. In the mandibular arch, lack of space was noted (Figure 1).

#### 2.2.3. Dental Cast Assessment

**Figure 2:**
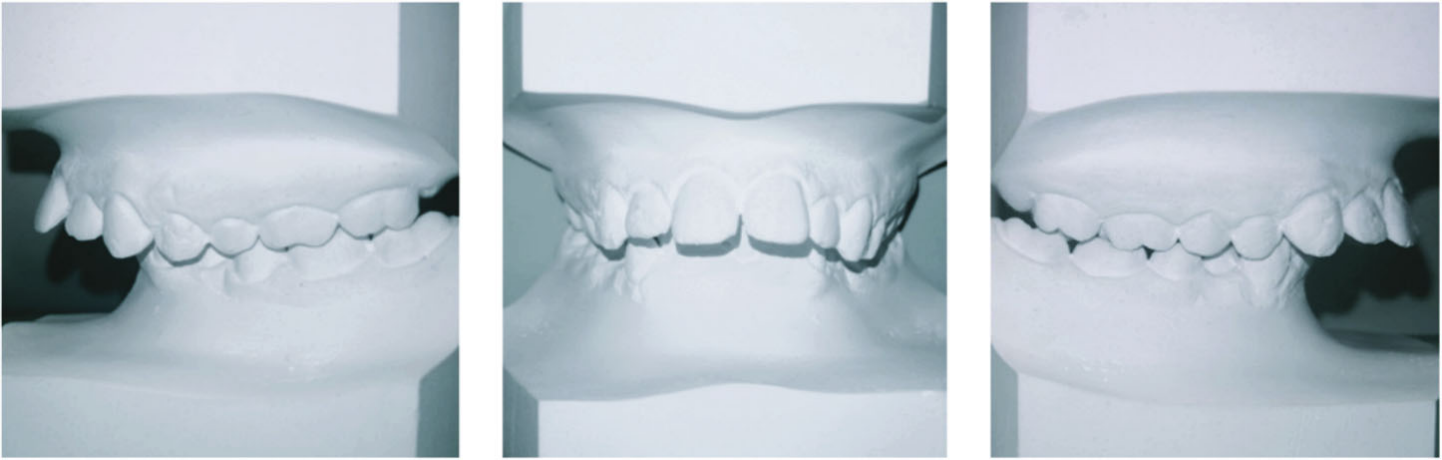
Dental cast models (T0); T0=pretreatment.

The dental cast analysis also showed an increased OVJ and OVB and enhanced Spee's curve (Figure 2).

#### 2.2.4. Radiographic Assessment

Radiographically, a complete permanent dentition with unerupted third molars was observed (Figure 1).

**Figure 3:**
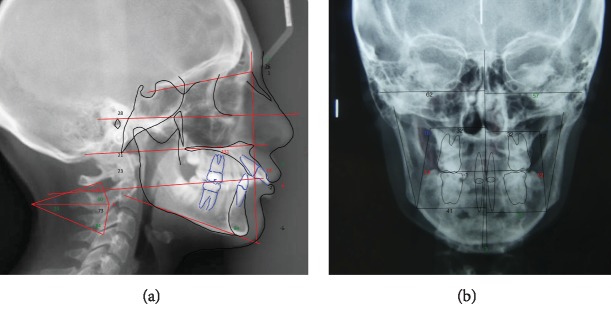
Cephalometric tracing (T0): (a) cephalometric evaluation of the lateral radiograph of the skull and (b) cephalometric evaluation of the posteroanterior radiograph of the skull; T0=pretreatment.

The cephalometric evaluation of the lateral radiograph of the skull (Figure 3(a); Table 1, T0) showed that the patient had a skeletal Class II pattern (Wits: 5.7 mm) with a slightly retruded position of the maxilla (SNA: 77.1°) and mandible (SNB: 75.8°). The occlusal plane was significantly anteriorly upward pitched (Mand plane-Occ plane: 23.6°).

The maxillary incisors were protruded (A1 to APo plane: 2.3 mm) and proclinate (U1-Occ plane: 50.0°; U1-palatal plane: 131.0°), and the mandibular incisors were slightly retruded (L1 to Apo plane: 12.4 mm) and reclined (IMPA: 83.5°).

The cephalometric evaluation of the skull radiograph in the posteroanterior (PA) view showed good symmetry overall (Figure 3(b)).

#### 2.2.5. Diagnosis

No symptoms of TMJ dysfunction were referred (there were neither noises from the joint nor pain identified during mandibular movement; there was a good range of mandibular movement).

The patient was diagnosed as having a skeletal Class II pattern with dental deep bite and severe dental Class II combined with dental crowding, in particular in the anterior part of the lower arch. The accentuated Spee's curve, resulting from an overeruption of lower incisors in association with the anterior labial flaring of the upper incisors, led to the tilting of the occlusal plane anteriorly upward.

### 2.3. Treatment

#### 2.3.1. Treatment aim

The following treatment objectives were established:

1. To obtain space in the arches to accommodate the teeth
2. To expand the maxillary arch to obtain harmony with the mandibular arch
3. To expand the mandibular arch
4. To reduce OVJ and OVB
5. To correct molar and canine Class II dental malocclusion
6. To restore lip competence at rest and to reduce the mentalis habit
7. To improve facial and smile aesthetics reducing buccal corridors

The expected compliance was good.

#### 2.3.2. Treatment Plan

Based on the diagnostic records and consultation with the patient, the following plan was developed:

1. Extraction of 1.8, 2.8, 3.8, and 4.8
2. Surgically Assisted Rapid Palatal Expansion (SARPE) using bone-borne transpalatal distractor (TPD) and MMD using bone-borne transmandibular distractor (TMD).
3. Comprehensive orthodontic treatment with particular attention paid to anterior teeth position and relationship
4. Surgical planning reevaluation of the orthodontic results
5. Corrective combined jaw surgery: usual bimaxillary advancement procedure with deliberate counterclockwise rotation of the occlusal plane including LeFort I osteotomy of the maxilla for anterior maxillary impaction and advancing; Bilateral Sagittal Split Osteotomy (BSSO) for increasing lower jaw length to provide better balance to the facial features
6. Postsurgical orthodontics and periodontal follow-up

#### 2.3.3. Treatment Progress

After the removal of the wisdom teeth, a Surgically Assisted Rapid Palatal Expansion using bone-anchored TPD and a Surgically Assisted Mandibular Midline Distraction procedure using bone-anchored TMD were performed.

Both surgical procedures were carried out under general anesthesia with nasotracheal intubation.

The surgical technique and distraction protocol used for SARPE was performed according to Ramieri et al. [19].

A flattened horseshoe-shaped incision was executed high in the vestibule from approximately the second premolar to the second premolar ensuring excellent exposure of the osteotomy site. The surgical cutting of the bone was performed from the piriform aperture to the pterygomaxillary suture, approximately 8 mm above the apices of the teeth. A vertical corticotomy from the anterior nasal spine to the alveolar crest was made using a surgical bur. The maxilla was downfractured sagittaly using a thin, straight osteotome. Pterygomaxillary suture disjunction was obtained using curved osteotomes.

A bone-borne distraction device (TPD™, Surgi-Tec NV, Brugge, Belgium) was subsequently placed to the palatal vault, through small mucosal incisions nearby second premolars, and fixed with 7 mm screws.

The surgical technique used for Surgically Assisted Rapid Mandibular Expansion was performed according to Mommaerts et al. [4]. A labial vestibular incision was carried out in order to gain access for the step osteotomy, consisting of two vertical osteotomies (interdental between canine and later incisor; in the basal bony midline) connected by a horizontal subapical osteotomy. A bone-borne distraction device (TMD™, Surgi-Tec NV, Bruges, Belgium) was subsequently placed on the mandibular symphysis.

The whole surgical procedure had taken approximately 80 minutes.

The patient was hospitalized for one night and was discharged the next morning with antibiotic, analgesic, and corticosteroid therapy. No complication after surgical procedures was reported.

After seven days, the maxillary distractor was activated (0.33 mm/day for the first ten days; 0.66/day thenceforth for an additional seven days); concomitant activation of the mandibular distraction device was performed (2 × 0.5 mm/day for ten days).

After starting the expansion procedure, the amount of the widening was clinically evaluated weekly: the spacing between the maxillary central incisors, the mandibular lateral incisor, and canines was the first sign of disjunction of the bone halves.

Total expansion of 8 mm, assessed by measuring the maxillary intermolar width as the distance between the mesial fossae of the maxillary first molars, was achieved in the upper arch. Total expansion of 5 mm, assessed by measuring the mandibular intercanine width as the distance from cusp tip to cusp tip of the mandibular canines, was achieved in the lower arch.

**Figure 4:**
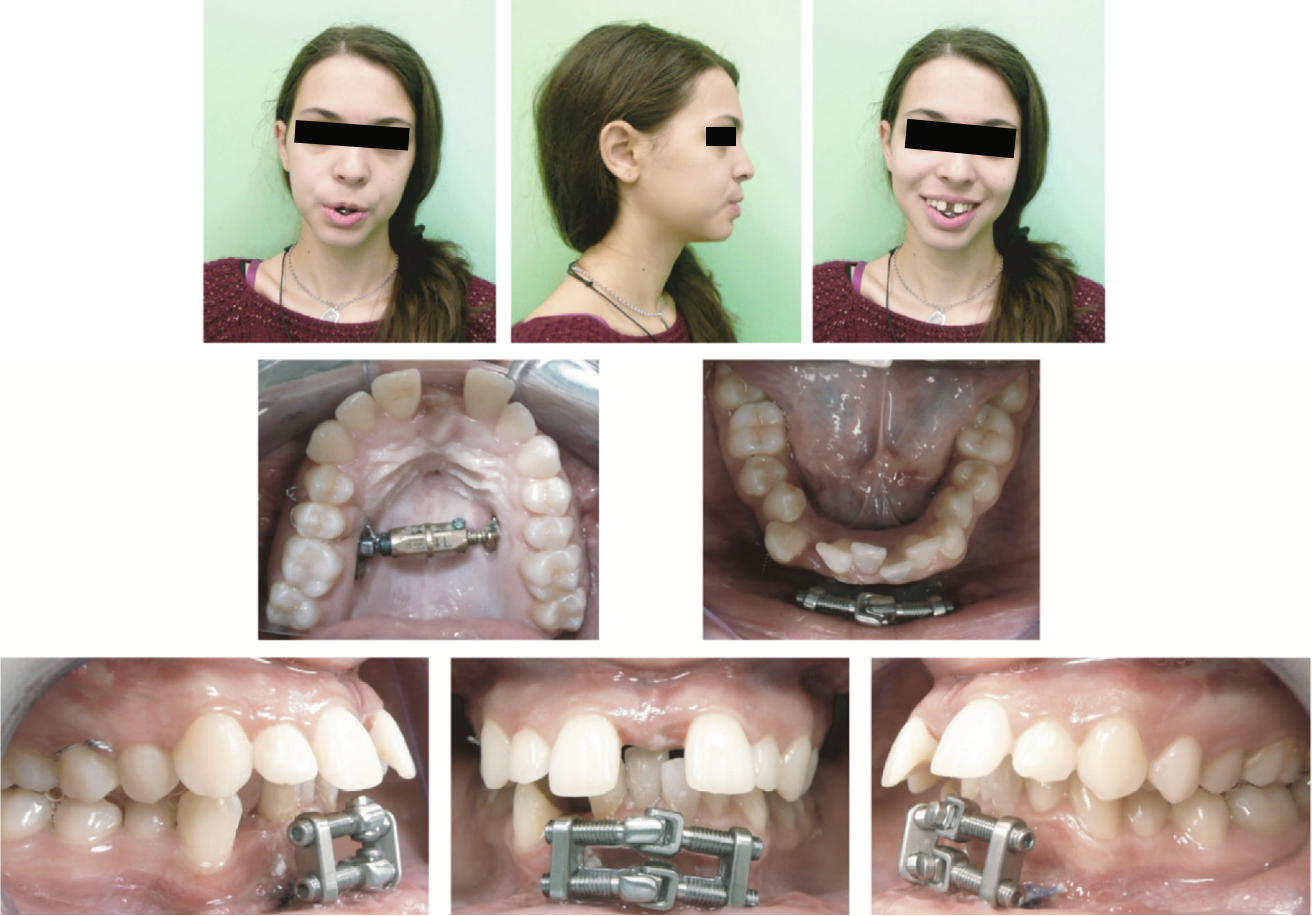
Extraoral photographs (T1) and intraoral photographs (T1); T1=treatment progress (1 month).

After the postoperative activation phase, both expansion devices were locked with a securing screw to prevent their deactivation (Figure 4) and to ensure the consolidation phase.

The patient's clinical status was assessed 15 days after expansion in progress: no teeth mobility, no gingival recession, no periodontal pockets, unaltered teeth vitality, and good hygiene with a suitable plaque without bleeding on probing control were detected.

After the surgically assisted widening of both the maxilla and the mandible, a fixed appliance (SW Roth prescription, 0.022^″^ × 0.028^″^ slots) was used to align the dentition during the consolidation phase. Before starting the active orthodontic treatment, a segmented arch-wire was used to control undesired movements of the lower incisors.

Both the distractors were removed under local anesthesia: the mandibular one was removed after about 120 days, and the maxillary one was removed after about 180 days. The orthodontic treatment continued following these stages:

1. *Aligning stage*: a lingual arch was mounted mandibulary after removing the upper and lower distractors to control the intermolar width and rotations. In both arches, .012 NiTi archwires were used. During this stage, an active coil was positioned between 35 and 33 and between 45 and 43 to create space for 34 and 44
2. *Levelling stage*: a TPB was mounted maxillary. NiTi archwires of successive cross-sections were used for about ten months in order to complete the alignment
3. *Working stage*: the maxillary and mandibular arches were coordinated, the maxillary and mandibular midlines were aligned, spaces were closed, and the maxillary and mandibular occlusal planes were paralleled. The OVJ and OVB were improved

**Figure 5:**
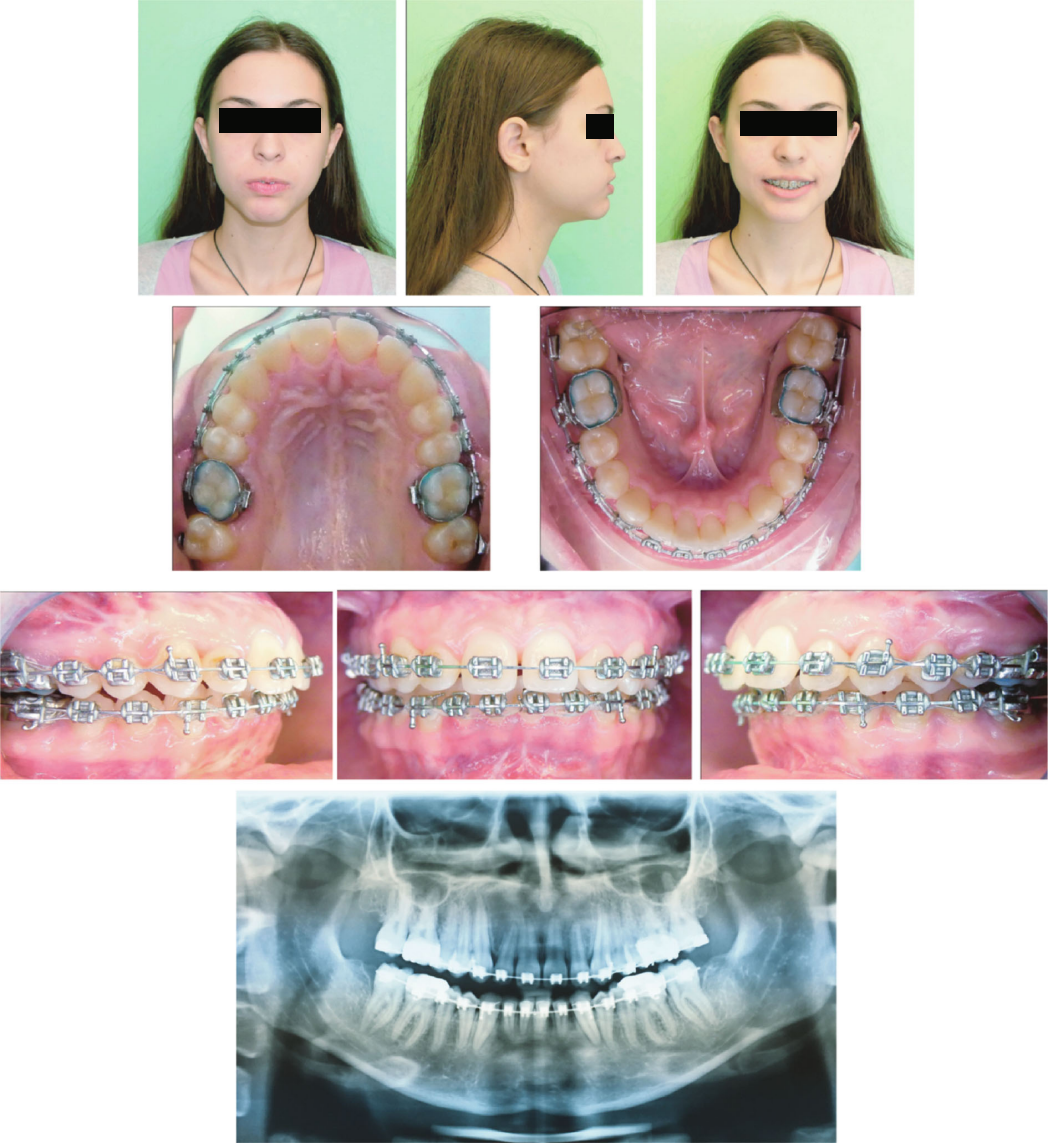
Extraoral photographs (T2), intraoral photographs (T2), and orthopantomogram (T2); T2=treatment progress (18 months).

A panoramic radiograph to evaluate the position of the roots and a lateral radiograph of the skull that showed a clockwise rotation of the maxillomandibular complex were taken. Also, the clinical examination showed excessive incisal exposure during the smile (Figure 5).

Presurgical orthodontic results were reevaluated together with the orthognathic surgery team of the University of Turin to decide the proper treatment plan.

In order to improve aesthetics (correction of excessive tooth exposure at rest and increase of soft tissue support with skeletal facial expansion) and improve dental and occlusal relationship, maxillomandibular osteotomies were planned with the agreement of the patient. A total of 4 mm of maxillary impaction and 7 mm of mandibular advancement were programmed. The following corrective combined jaw surgery was performed: LeFort I osteotomy of the maxilla with differential maxillary impaction and advancing simultaneously with a *Bilateral Sagittal Split Osteotomy* (BSSO), achieving a slight counterclockwise rotation of the occlusal plane.

**Figure 6:**
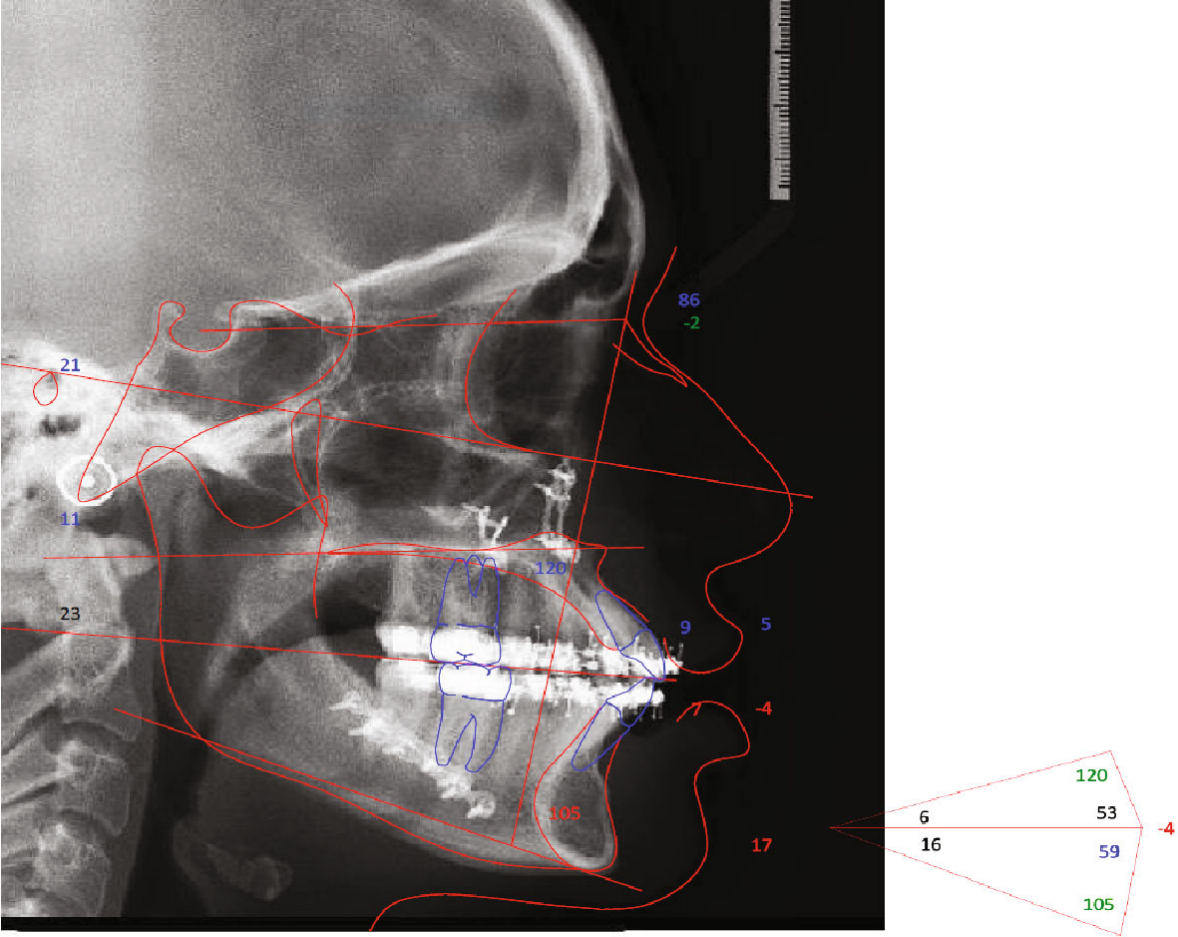
Cephalometric tracing (T2); T2=treatment progress (18 months).

A lateral radiograph of the skull was requested to do tracing for cephalometric evaluation: comparison of the cephalometric analysis before and after surgery demonstrated an improvement in the sagittal skeletal and dental values (Figure 6; Table 1, T2).

(4) *Detailing and finishing stage*: after surgery, the patient was monitored closely for one month and was then referred for postsurgical orthodontics. At that point, with the same archwires placed, she wore light posterior vertical elastics and bilateral II class elastics full time for three months. The postoperative stage was aimed at additional finalization; therefore, coordination of the maxillary and mandibular arches were followed by finishing and detailing of the occlusion

It was decided that the treatment goals were achieved, and active treatment could be completed. Approximately 30 months spread out over 62 appointments was the comprehensive active treatment time.

### 2.4. Summary

#### 2.4.1. Treatment Results

On the day of debonding, extra- and intraoral photographs were taken along with impressions and occlusion wax to build the final plaster models. For the retention, it was decided to use a Hawley appliance for the upper teeth and a bonded retainer for the lower teeth.

**Figure 7:**
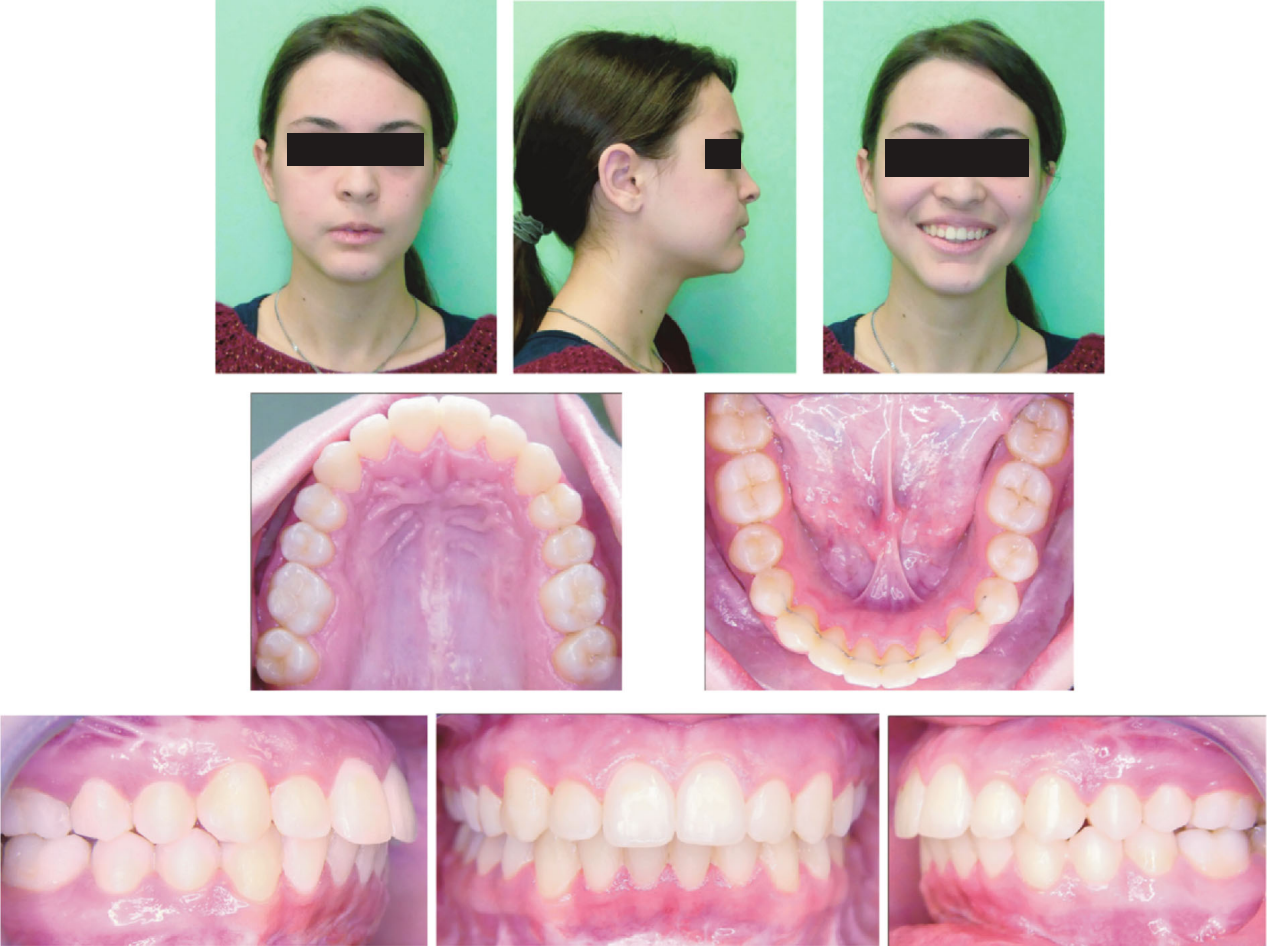
Extraoral photographs (T3) and intraoral photographs (T3); T3=posttreatment (36 months).

Review of the treatment outcome with clinical examination, extra- and intraoral photographs (Figure 7), and the final plaster models showed the following outcomes:

- Mandibular symmetrization, improvement of smile arch aesthetic, reduction of buccal corridors, and an improved lower third projection
- Correction of the dental Class II was achieved
- Correction of arch shapes was observed
- Significant reduction of the lip incompetence was obtained
- Mentalis habit was reduced

#### 2.4.2. Follow-Up

**Figure 8:**
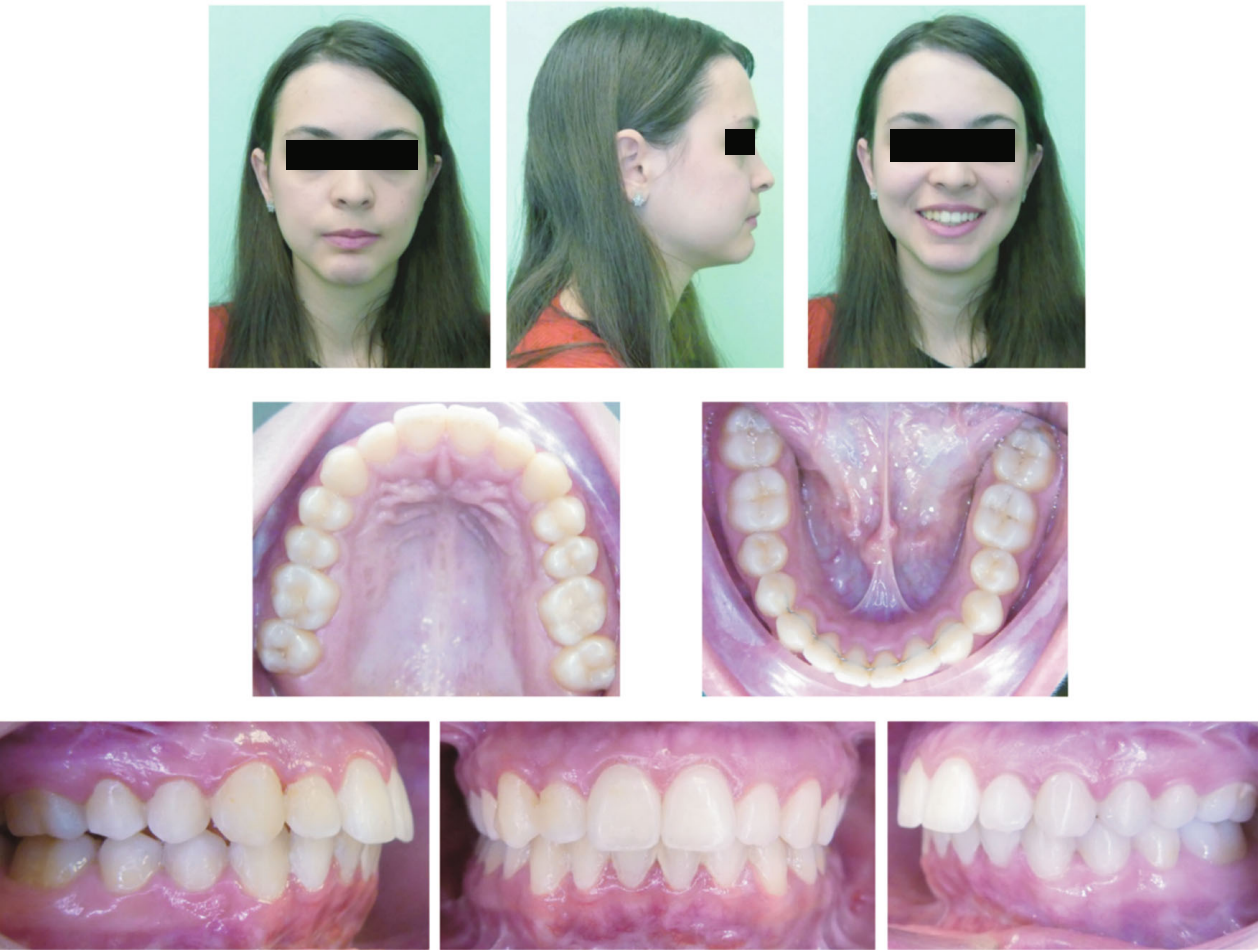
Extraoral photographs (T4) and intraoral photographs (T4); T4=follow-up (30 months after the end of the treatment).

**Figure 9:**
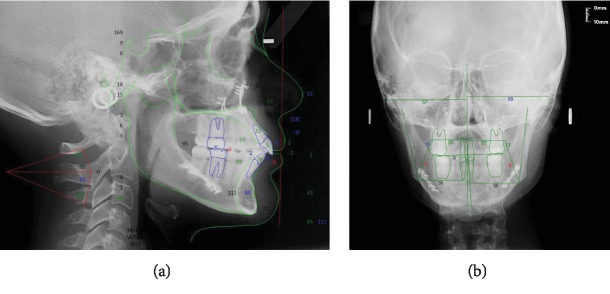
Cephalometric tracing (T4): (a) cephalometric evaluation of the lateral radiograph of the skull and (b) cephalometric evaluation of the posteroanterior radiograph of the skull; T4=follow-up (30 months after the end of the treatment).

Approximately 30 months after debonding, the shapes of the arches and occlusion appeared to be stable, even though a moderate amount of relapse occurred (Figure 8). Both cephalometric evaluations of the lateral radiograph of the skull (Figure 9(a); Table 1, T4) and the posteroanterior radiograph of the skull (Figure 9(b)) showed a nonsignificant difference compared to postsurgery assessment except for sagittal jaw relationship that exhibited a considerable relapse (Table 1, T2 and T4).

## 3. Discussion

In selected adult patients, the simultaneous osteodistraction of both the maxilla and mandible represent a viable option to obtain satisfactory and stable outcomes.

The basic requirements for an optimal result of osteodistraction are minimally traumatic osteotomies, stable mechanical fixation across osteotomies, an adequate latency period before lengthening (to establish repair processes), suitable rhythm and amplitude of lengthening, a realistic goal regarding the extent of lengthening, and sufficient time for the callus to mature before frame or nail removal [20].

The use of proper appliances, careful surgery and follow-up, and cooperation between the surgical and orthodontic teams are the key to success in this demanding approach.

Discomfort, apical trauma, and local infections are the main disadvantages of TMD, as reported in the literature [4, 21].

Lip and cheek irritation, as well as speech and eating difficulties are commonly related to osteodistraction devices. Gingival recession, gingivitis, and irritation of the mucosa often represent soft tissue complications [22, 23].

Concerning the present case, several treatment alternatives were explored. Nevertheless, dental compensation could not be achieved because severe crowding in the lower arch cannot be properly fixed with Interproximal Enamel Reduction (IPR) and inclination of the mandibular incisors. As an alternative, the extraction of one mandibular central incisor could be considered.

Therefore, TMD was the best option for mandibular expansion and arch form improvement.

Concerning the upper arch, the severe inclination of the maxillary incisors reduces the chances to solve the crowding only with IPR.

Anterior tooth retraction after molar distalization and retraction using temporary anchorage devices (such as a zygoma anchor device [24] or palatally located temporary anchorage device [25]) might have been an alternative treatment option in order to mitigate the severe overjet and to secure an enhanced molar and canine relationship.

However, the narrow shape of both arches would be left unaltered.

Therefore, SARPE was the best option for maxillary arch expansion and arch form improvement.

Extraction of two bicuspids in both arches was aimed at obtaining sufficient space to correct the severe crowding and to increase OVJ properly; this treatment option was rejected because this would compromise both the occlusion, the profile, and the dental aesthetics.

Overall, even though some care must be considered, MMTOD can be recognized as a safe form of treatment.

As regards the type of distractors that can be used, they can be divided into tooth-borne, bone-borne, and hybrid devices, mainly differing from the position of the fixation points and the stiffness of the appliance.

Tooth-borne devices induce less parallel expansion than bone-borne distractors because they apply their vector above the center of resistance: this difference implies an increase of anterior mandible widening when using the tooth-borne devices as compared when using the bone-borne and hybrid distractors.

The parallel expansion of the hemimandibles [26] leads to bone regeneration both at the basal and alveolar level, and it is crucial to reduce relapse.

Ideally, a basal bone widening would decrease the long-term relapse [27, 28].

The rigidity of a distractor defines the parallel movement of segments and the quantity of micromotion between segments.

The choice of bone-borne appliances is preferable because of a biomechanical advantage (vector applied close to the center of resistance) and a clinical advantage (smaller loss of anchorage and less skeletal relapse both during and after expansion and lower incidences of cortical fenestration and buccal root compared to tooth-borne distractors [29]; reduced risk of craniomandibular disorders minimizing lateral displacement of the condyle [30]).

In this case, the enlargement of the transversal diameter was obtained from the canines to the first and second premolars leading to a “fan-shaped” maxilla.

The symphyseal osteodistraction demonstrated parallel movements along the lower jaw, leading to a “square-shaped” mandible.

The slight bimaxillary advancement was deemed necessary to achieve completely the aesthetic goals of the treatment completely, with operational control of facial height and projection.

We decided to increase the facial projections with a slight undercorrection of the facial height in order to leave the patient's face slightly long from a qualitative standpoint, with the elimination of the lip strain and an attractive smile with the elimination of buccal corridors because of the previous maxillomandibular transversal expansion.

The Hawley retainer for the upper arch was selected to promote postorthodontic settling of the posterior occlusion of the patient [31]; this is a bonded retainer for the lower arch because of the slightly better stability over removable retainers and more acceptability to wear. A multistranded stainless steel wire was preferred over a fiber-reinforced composite retainer because of the absence of aesthetic concerns or allergy to metals [32].

The follow-up showed satisfactory long-term stability even though significant skeletal relapse was reported in the literature [33].

## 4. Conclusions

The Maxillomandibular Transverse Osteodistraction technique associated with orthognathic surgery described here could obtain both functional and aesthetic results in patients with reduced maxillary and mandibular transverse dimensions and severe dental crowding.

## Figures and Tables

**Table 1:** Cephalometric analysis (T0-T2): T0=pretreatment, T2=treatment progress (18 months), and T4=follow-up (30 months after the end of the treatment).

	Measured value T0	Measured value T2	Measured value T4	Norm
SNA	**77.1**°	**83.6**°	***83.1***°	82° ± 3.5°
SNB	**75.8**°	**86.1**°	***80.9***°	80° ± 3°
ANB	**1.4**°	**-2.6**°	***2.2***°	2° ± 2.4°
Maxillary skeletal (A-Na Perp)	-**3.0**°	**5.1**°	***1.6***°	0° ± 3.1°
Mand. skeletal (Pg-Na Perp)	-**5.0**°	**16.5**°	***1.1***°	0° ± 5.3°
Wits appraisal	**5.7** mm	**-4.2** mm	***-0.1***°	0 *mm* ± 1 *mm*
FMA (MP-FH)	**21.1**°	**10.9**°	***20.7***°	26° ± 5°
MP-SN	**30.6**°	**23.3**°	***29.2***°	33° ± 6°
Palatal-Mand angle	**22.7**°	**22.8**°	***23.0***°	28° ± 6°
Palatal-Occ plane	-**1.0**°	**6.3**°	***7.0***°	10° ± 4°
Mand plane-Occ plane	**23.6**°	**16.5**°	***16.0***°	17.4° ± 5°
U1 protrusion (U1-APo)	**2.3** mm	**8.9** mm	***8.6*** mm	0.4 ± 2.0 *mm*
L1 protrusion (L1-Apo)	**12.4** mm	**7.4** mm	***4.5*** mm	3.5 ± 2.3 *mm*
U1-palatal plane	**131.0**°	**120.2**°	***118.4***°	110° ± 5°
U1-Occ plane	**50.0**°	**53.4**°	***54.6***°	57.5° ± 7°
L1-Occ plane	**72.9**°	**58.6°**	***60.6°***	72° ± 5°
IMPA	**83.5**°	**105.0°**	***103.4°***	95° ± 7°

## Abbreviations

MSDO: Mandibular symphyseal distraction osteogenesis
MMD: Mandibular midline (osteo-) distraction
TMSD: Transmandibular symphyseal (osteo-) distraction
SARPE: Surgically assisted rapid palatal expansion
SARME: Surgically assisted rapid maxillary expansion
MMTOD: Maxillomandibular transverse osteodistraction
CCE: Common childhood exanthemas
TMJ: Temporomandibular joint
OVJ: Overjet
OVB: Overbite
TPD: Transpalatal distractor
TMD: Transmandibular distractor
BSSO: Bilateral sagittal split osteotomy
IPR: Interproximal enamel reduction.

## References

[1] Gandini P., Schiavi A., Camassa D., Manuelli M. (1989). Statistical survey of malocclusion in school age children. *Mondo Ortodontico*.

[2] Bayram M., Ozer M., Arici S., Alkan A. (2007). Nonextraction treatment with rapid maxillary expansion and mandibular symphyseal distraction osteogenesis and vertical skeletal dimensions. *The Angle Orthodontist*.

[3] Malkoç S., Üşümez S., Işeri H. J. A. J. (2007). Long-term effects of symphyseal distraction and rapid maxillary expansion on pharyngeal airway dimensions, tongue, and hyoid position. *American Journal of Orthodontics and Dentofacial Orthopedics*.

[4] Mommaerts M. Y., Spaey Y. J. E., Correia P. E. G. S., Swennen G. R. J. (2008). Morbidity related to transmandibular distraction osteogenesis for patients with developmental deformities. *Journal of Cranio-Maxillofacial Surgery*.

[5] Burstone C. (1989). Precision lingual arches. Active applications. *Journal of Clinical Orthodontics*.

[6] McDougall P. D., McNamara J. A., Dierkes J. M. (1982). Arch width development in Class II patients treated with the Frankel appliance. *American Journal of Orthodontics*.

[7] Weinberg M., Sadowsky C. (1996). Resolution of mandibular arch crowding in growing patients with Class I malocclusions treated nonextraction. *American Journal of Orthodontics and Dentofacial Orthopedics*.

[8] Pascon L., Bazert C., Bardinet E. (2016). Contribution of mandibular symphyseal distraction osteogenesis to our therapeutic strategies. *Journal of Dentofacial Anomalies and Orthodontics*.

[9] Sperber G. J. C. D. H. (2001). *Facial Skeleton*.

[10] King J. W., Wallace J. C., Winter D. L., Niculescu J. A. (2012). Long-term skeletal and dental stability of mandibular symphyseal distraction osteogenesis with a hybrid distractor. *American Journal of Orthodontics and Dentofacial Orthopedics*.

[11] Guerrero C., Bell W., Contasti G., Rodriguez A. (1997). Mandibular widening by intraoral distraction osteogenesis. *British Journal of Oral and Maxillofacial Surgery*.

[12] Haas A. (1980). Long-term posttreatment evaluation of rapid palatal expansion. *The Angle Orthodontist*.

[13] Brown G. (1938). *The Surgery of Oral and Facial Diseases and Malformations*.

[14] Betts N. J., Vanarsdall R. L., Barber H. D., Higgins-Barber K., Fonseca R. J. (1995). Diagnosis and treatment of transverse maxillary deficiency. *The International Journal of Adult Orthodontics and Orthognathic Surgery*.

[15] Suri L., Taneja P. (2008). Surgically assisted rapid palatal expansion: a literature review. *American Journal of Orthodontics and Dentofacial Orthopedics*.

[16] King J. W., Wallace J. C. (2004). Unilateral Brodie bite treated with distraction osteogenesis. *American Journal of Orthodontics and Dentofacial Orthopedics*.

[17] Proffit W. R., White R. P., Sarver D. M. (2003). *Contemporary Treatment of Dentofacial Deformity*.

[18] Bianchi F. A., Gerbino G., Corsico M. (2017). Soft, hard-tissues and pharyngeal airway volume changes following maxillomandibular transverse osteodistraction: computed tomography and three-dimensional laser scanner evaluation. *Journal of Cranio-Maxillofacial Surgery*.

[19] Ramieri G. A., Spada M. C., Austa M., Bianchi S. D., Berrone S. (2005). Transverse maxillary distraction with a bone-anchored appliance: dento- periodontal effects and clinical and radiological results. *International Journal of Oral and Maxillofacial Surgery*.

[20] AI-Aql Z. S., Alagl A. S., Graves D. T., Gerstenfeld L. C., Einhorn T. A. (2008). Molecular mechanisms controlling bone formation during fracture healing and distraction osteogenesis. *Journal of Dental Research*.

[21] von Bremen J., Schäfer D., Kater W., Ruf S. (2008). Complications during mandibular midline Distraction. *The Angle Orthodontist*.

[22] Alkan A., Özer M., Baş B. (2007). Mandibular symphyseal distraction osteogenesis: review of three techniques. *International Journal of Oral and Maxillofacial Surgery*.

[23] Gunbay T., Akay M. C., Aras A., Gomel M. (2009). Effects of transmandibular symphyseal distraction on teeth, bone, and temporomandibular joint. *Journal of Oral and Maxillofacial Surgery*.

[24] Cornelis M. A., de Clerck H. J. (2007). Maxillary molar distalization with miniplates assessed on digital models: a prospective clinical trial. *American Journal of Orthodontics and Dentofacial Orthopedics*.

[25] Nienkemper M., Wilmes B., Pauls A., Yamaguchi S., Ludwig B., Drescher D. (2014). Treatment efficiency of mini-implant-borne distalization depending on age and second-molar eruption. *Journal of Orofacial Orthopedics / Fortschritte der Kieferorthopädie*.

[26] Raoul G., Wojcik T., Ferri J. (2009). Outcome of mandibular symphyseal distraction osteogenesis with bone-borne devices. *Journal of Craniofacial Surgery*.

[27] Conley R., Legan H. (2003). Mandibular symphyseal distraction osteogenesis: diagnosis and treatment planning considerations. *The Angle Orthodontist*.

[28] Del Santo M., Guerrero C. A., Buschang P. H., English J. D., Samchukov M. L., Bell W. H. (2000). Long-term skeletal and dental effects of mandibular symphyseal distraction osteogenesis. *American Journal of Orthodontics and Dentofacial Orthopedics*.

[29] Mommaerts M. (1999). Transpalatal distraction as a method of maxillary expansion. *British Journal of Oral and Maxillofacial Surgery*.

[30] Mommaerts M. Y., Polsbroek R., Santler G., Correia P. E. G. S., Abeloos J. V. S., Ali N. (2005). Anterior transmandibular osteodistraction: clinical and model observations. *Journal of Cranio-Maxillofacial Surgery*.

[31] Bauer E. M., Behrents R., Oliver D. R., Buschang P. H. (2010). Posterior occlusion changes with a Hawley vs Perfector and Hawley retainer: a follow-up study. *The Angle Orthodontist*.

[32] Lucchese A., Manuelli M., Bassani L. (2015). Fiber reinforced composites orthodontic retainers. *Minerva Stomatologica*.

[33] Rossini G., Vinci B., Rizzo R., Pinho T. M. D., Deregibus A. (2016). Mandibular distraction osteogenesis: a systematic review of stability and the effects on hard and soft tissues. *International Journal of Oral and Maxillofacial Surgery*.

